# Operational Insights into Analysing Team and Player Performance in Elite Rugby League: A Narrative Review with Case Examples

**DOI:** 10.1186/s40798-022-00535-7

**Published:** 2022-12-03

**Authors:** Corey James Wedding, Carl Thomas Woods, Wade Heath Sinclair, Anthony Scott Leicht

**Affiliations:** 1 Sport and Exercise Science, James Cook University, Townsville, QLD Australia; 2 North Queensland Cowboys Rugby League Football Club, Townsville, QLD Australia; 3 Institute for Health and Sport, Victoria University, Melbourne, Australia

**Keywords:** Rugby League, Data analysis, Performance analysis, Team sport, Sports technology

## Abstract

In professional team sports, like Rugby League, performance analysis has become an integral part of operational practices. This has helped practitioners gain deeper insight into phenomena like team and athlete behaviour and understanding how such behaviour may be influenced by various contextual factors. This information can then be used by coaches to design representative practice tasks, inform game principles and opposition strategies, and even support team recruitment practices. At the elite level, the constant evolution of sports technology (both hardware and software) has enabled greater access to information, making the role of the performance analyst even more valuable. However, this increase in information can create challenges regarding which variables to use to help guide decision-making, and how to present it in ways that can be utilised by coaches and other support staff. While there are published works exploring aspects of performance analysis in team sports like Rugby League, there is yet to be a perspective that explores the various operational uses of performance analysis in Rugby League, the addition of which could help guide the practices of emerging performance analysts in elite organisations like the Australian National Rugby League and the European Super League. Thus, this narrative review—with accompanying case examples—explores the various ways performance analysis can help address pertinent operational questions commonly encountered when working in high-performance sport.

**Supplementary Information:**

The online version contains supplementary material available at 10.1186/s40798-022-00535-7.

## Key Points

- Data reduction and clustering, logistic regression and decision support analysis can each play an important part in supporting sports performance analysts.
- The exemplars provided are intended to offer guidance as to how various analytical techniques may be used to address key questions commonly encountered in high-performance sport.

## Introduction

Sports performance analysis is an increasingly important part of operational practices in high-performance sport [1–3]. So much so, that many professional organisations now staff positions with full-time specialists and at times, form entire departments. These are roles which are increasingly supported by the growing tertiary offerings of postgraduate degrees in sports performance analytics. Yet, despite the nuances of these positions, the role of the performance analyst in high-performance sport is often diverse, ranging from assisting practitioners with questions stemming from practice task design [4, 5], team strategies in competition (i.e. the development and monitoring of game principles) [6–9], and team selection and recruitment [10–12], to exploring the efficacy of long-term performance gains associated with various training interventions [13, 14]. Such operational insights can also span multiple developmental levels (i.e. junior-to-senior transition), offering insight into individual, team or competition-wide behaviours that change over varying timescales (i.e. within or across seasons) [15, 16].

There is, however, a trade-off associated with this growing operationalism of performance analysis in high-performance sport. Notably, there is an increased strain on support staff to analyse and present data in meaningful and actionable ways for coaches, athletes, and other practitioners [2, 16, 17]. Magnifying this challenge, there is little empirical guidance that supports performance analysts working in high-performance sport when navigating the varying methods available for the analysis of the ever-growing sea of data, fuelled by the rise of sports technology [18]. This, in part, could be due to the diverse questions and problems that performance analysts in high-performance sport are often asked to assist with—demanding a range of adaptable skillsets [17]. Whilst the introduction of various graduate courses and certificates in sports performance analytics at tertiary institutions has begun to support the next wave of performance analysts,^1^ there is little information available for those already working within professional sport.

In light of this, our aim is to present a sample of techniques that could be of use for developing performance analysts, primarily focused on the team sport of Rugby League. This does not intend to cover an exhaustive set of analytical techniques, but rather focuses on certain ones that could be of assistance to developing performance analysts in Rugby League, associated with common questions asked by coaches at varying levels of competition. The paper is thus set out in two parts. The first reviews techniques related to data reduction and clustering, decision trees, and logistic regression. In the second, two case examples that demonstrate each technique in practice are presented. The goal of this second part is to act as a means of demonstration, guiding developing performance analysts in how they may employ such techniques, rooted in real-world questions. Thus, the questions posed in these case examples are questions which the first author of this paper, who is currently working as a performance analyst for a professional Rugby League team, has had to navigate. So, what is ‘out there’ for developing sports performance analysts interested in individual and team performance in high-performance sport?

## Part 1: An Overview of Certain Techniques for Sports Performance Analysts

### Performance Analysis Practices in Team Sports

Like any high-performance environment, successful performance in elite-level sport requires skilled functionality, such as working out ways to offload the ball in Rugby League [19], or ways of serving to various regions of a tennis court to exploit opponent positioning [20]. These sports-specific functional components are often referred to as ‘technical skills’ [21] and are typically captured by performance analysts to help coaches understand various aspects of game play as it unfolds. For example, capturing and analysing information related to how a player obtains and then disposes of the ball in Australian football (AF) can assist coaches with the design of training activities intended to promote the development of offensive behaviour [13, 22]. Further, application of similar notational analyses at a team level could lead to information that resolves collective behaviour—manifest in styles or common patterns of play—which can be modelled relative to outcomes like match success. This is noted in the work of Lago-Peñas et al. [23], who identified five factors (i.e. groups of performance indicators) that explained various styles of play across an elite soccer competition, information which they argued could be strategically used by coaches to counter an opposition. As we now go onto discuss, an integral component of the analysis used by Lago-Peñas et al. [23] was data reduction and clustering—whereby large multidimensional datasets were reduced to factors and clustered based on their similarity, allowing practitioners to make decisions with reference to a select few (important) variables [8, 24, 25].

### Data Reduction and Clustering

While sports technology has unquestionably assisted performance analysts [3], it has resulted in a large quantity of data to be filtered, analysed, and reported in actionable ways [15, 26]. This has likely led to uncertainty with regard to variable selection—defined as which variables (or groups of variables) are important in supporting practitioners in making decisions guided by sports performance data [15, 27]. In light of this, performance analysts have sought to apply various data reduction techniques—common to other quantitative disciplines [27–29]—to hone in on (combinations of) performance indicators most important for explaining an outcome of interest [15, 16, 30]. In its broadest sense, data reduction is a process by which large—often multidimensional—datasets can be reduced into smaller, more manageable sets, while ensuring the integrity of the data is not compromised [26]. In high-performance sport where the quantity of data is expanding given the automation of various sports technologies, such reduction techniques can be vitally important.

While there are a variety of data reduction techniques, two of the more common seen in team sports, like Rugby League, are *principal component analysis* and *multidimensional scaling* [25, 31, 32]. Both principal component analysis and multidimensional scaling produce a series of factors which represent groups of similar variables [33–35]. These techniques differ, though, with respect to the processes involved with the creation of these factors. For example, principal component analysis resolves linear, uncorrelated sets of variable combinations—achieved by resolving the eigenvalue, a scaling factor which determines the magnitude and number of principal components (factors) to be used [26, 33, 36]. Conversely, multidimensional scaling relies on nonparametric regression to determine a dissimilarity ranking matrix to produce a series of dimensions, iteratively searching for least squares fit based on the rank order of the dissimilarities [25, 34, 37]. The rank order of dissimilarities and subsequent factors obtained via principal component analysis can then be used to explain various aspects of performance, such as what performance indicators are important for winning a match of Rugby League [8, 31, 38].

But how (or why) might we choose to use one technique over another? The key characteristics in each of these analyses are important to consider prior to selecting and utilising one over the other. To exemplify, as principal component analysis assumes a linear relationship within the data and the latent variables represented as factors, applying this technique to a nonlinear dataset may struggle to appropriately represent the distance measures between factors. Multidimensional scaling, on the other hand, assumes nonlinearity and strives only to optimise the fit between the dissimilarity of objects and the rank order of dissimilarities. Thus, understanding dataset properties is an important initial step in determining which technique is most appropriate in reducing its multidimensionality for sports performance analysts.

The use of these data reduction techniques has grown within Rugby League research. Notably, Woods et al. [25] highlighted the utility of multidimensional scaling for explaining the evolution of game play within the Australian National Rugby League over an 11-year period. These authors reduced a multidimensional dataset (dataset containing multiple different variables), visualising the ranked dissimilarities to show how the game evolved in a ‘follow-the-leader’ type manner (whereby the competition leaders evoke a successful style of play which other teams try to emulate in order to similarly succeed), postulating how coaches could use such insights to develop innovative styles or principles of play ‘beyond their time’. Comparatively, Parmar et al. [8] highlighted the utility of principal component analysis for the analysis of team performance in the European Super League. These authors identified that ‘making quick ground’, ‘quick play’, and ‘amount of possession’ were the most important factors for explaining match outcome [8]. Similarly, Wedding et al. [38] explored the use of principal component analysis for team performance analysis in the National Rugby League, identifying nine factors (six attacking, two defensive, and one contested) which could explain team playing styles relative to season and end of season rank—uncovering important characteristics for consideration in the design and implementation of game planning. Research in other sports such as soccer [23, 24], basketball [37, 39], and AF [40] has further exemplified the use of principal component and multidimensional scaling in identifying the performance characteristics most explanatory of team performance variance and playing style over varying time periods. Each of these studies demonstrates the value of data reduction in making actionably smaller subsets of data that maintains their underlying integrity. A further example of the utility of such a technique for servicing operational practices in Rugby League will be presented in the first Case Example*,* which is discussed in the second part of this review.

Clustering is another data reduction technique that is growing in popularity in sports performance analytics [41, 42]. A specific clustering technique discussed here is *two-step clustering*—a technique which reveals ‘natural’ clusters (or groupings) within a dataset using log-likelihood distance measures [5, 41, 43]. The utility of clustering for explaining phenomena in sport, like match outcome, has been exemplified by Gomez et al. [44] who grouped the performance of wheelchair basketball teams based on different match types (defined through score lines of ‘unbalanced’ or ‘balanced’). In being able to successfully cluster teams according to score lines, these authors demonstrated the use of this technique for reducing and visualising data into meaningful groups, which they argued was information important in supporting coaches to design game and practice strategies [44]. Further, Zhang et al. [5] utilised two-step clustering to identify five different player profiles of professional basketballers using anthropomorphic, technical, and physical variables—thereby supporting recruitment and talent selection. As an important aside, this study demonstrated the use of two-step clustering for handling data of variable properties (i.e. categorical and continuous), which is particularly critical for high-performance sport given the diverse sources of data often available to performance analysts [41, 45]. The use of two-step clustering for examining positional performance in Rugby League has been exemplified by Wedding et al. [32], who identified six positional groups (as compared to four a priori)—enabling the establishment of player performance profiles for performance assessment, player development, and recruitment.

Whilst only a snapshot of the available work, these studies do highlight the benefit of various data reduction and clustering techniques for sports performance analysts in high-performance environments. Nonetheless, to further guide developing performance analysts in adopting these data reduction techniques, the second part of this narrative review weaves in a case example demonstrating their use in practice. Before this, however, we next explore the use of *decision support analysis* (specifically decision trees) for sports performance analysts—showing how such a technique can support coaches and other practitioners in understanding the (nonlinear) interaction between variables, and how these interactions relate with various outcomes of practical interest.

### Decision Support Analysis

Indeed, data reduction and clustering analyses are some of many increasingly adopted methods for understanding what ‘successful’ performances look like in high-performance sport [5, 8, 24]. However, to support coaches in modifying targeted features of a game style to increase the probability of attaining a successful outcome, *decision support analyses* can be useful. Broadly, decision support analysis can support a practitioner by sifting through large quantities of data to identify underlying interactions and their conditional control statements, with this information being used to ascertain the probabilities of certain outcomes occurring [17, 46, 47]. The probabilities of these outcomes occurring can be visually represented in various forms, like decision trees, which can be easily interpreted and presented to coaching staff [31, 48]—guiding, challenging, or informing decision making [31, 49].

A growing decision support analysis in sports performance analytics are decision trees [15, 50, 51]. As the name implies, decision trees are models of decisions grown from a root or parent node, which iteratively grow branches that visualise the interaction between key variables and their conditional statements, explaining the probability of a certain outcome [51]. There are two primary types of decision trees: classification and regression [52–54]. Whilst there are some similarities between them (namely that neither require data normalisation), there are some key differences related to how the data are differentiated, grown or split during the analysis [52, 54]. Specifically, these differences relate to the underlying growth algorithm of the tree [52–54], meaning that while decision trees can be a useful tool for analysts given their capability to visualise complex, nonlinear interactions between variables, it is important to understand the appropriateness of types based upon the question asked and data used to grow the model [51, 52]. For example, if wanting to explain a binary variable of interest (i.e. win or loss/home or away), a CART (classification and regression tree) method may be appropriate. Fernandes and colleagues [48] exemplified the use of CART as a method for explaining the likelihood of a passing or rushing play occurring at any point during a National Football League game. On the other hand, if seeking to explain a non-binary outcome, a CHAID (chi-squared automatic interaction detection) algorithm may be appropriate given that it utilises multi-way splits, which could be used to identify multiple styles or phases of play [31]. Not only are the number of splits that may occur from any given node different depending on which model is chosen, but so too is the way in which the model decides how to make these splits and when it decides to stop splitting [51–53]. Thus, understanding which tree to use is an important initial step for sports performance analysts—being implicated by the question seeking to be answered and the data used to answer it.

In team sports, decision trees have shown capability to explain complex interactions of performance indicators that contribute to match outcome in Australian football [9, 55], Rugby League [31, 45, 56], basketball [57, 58], and soccer [49]. Further, decision trees have been used to identify performance gaps between competition levels, with such information being critical to support talent development in sports like Rugby League [56, 59, 60]. Beyond team performance, decision support analysis has been used to explain player and playing position behaviours within team sports [5, 42, 61], with Morgan et al. [62] highlighting that attackers held a distinct advantage in one-on-one situations in hockey when moving at velocities ≥ 0.5 m s^−1^. However, in instances where the initial speed differential between attackers and defenders was small (< 0.5 m s^−1^), the attackers’ probability of winning the encounter could improve if defenders held a lateral speed > 1.4 m.s^−1^ [62]. This level of detail clearly supports practitioners and athletes in the design of practice tasks and establishment of various strategies intended to exploit opponents and gain a competitive advantage when coupled with their experiential knowledge. Thus, decision support analyses, like decision trees, are useful in high-performance sport, particularly regarding the identification of team performance indicators and their conditional control statements that lead to increased chances of attaining match success [9, 49, 57].

Successful application of these techniques could offer practitioners another way of analysing and visualising various interactions of key variables during a match—further supporting decisions around training and game-planning strategies. The case example detailed in the second part of this review exemplifies the practical utility of decision support analysis for the resolution of important team playing styles relative to playing at home or away within Rugby League. Prior to this, though, we next explore the use of *logistic regression* for sports performance analysts—highlighting how this technique could be implemented as another method to support coaches in understanding interactions that could exist within the various training and match data.

### Logistic Regression

So far, this review has examined the efficacy of data reduction, clustering, and decision support analysis for the exploration of important technical and tactical characteristics in high-performance sport. Logistic regression is a technique used to exclusively model the probability of a dichotomous event (e.g. win or loss) occurring whilst accounting for one or more independent variables that influence the event [8, 58, 63]. There are many benefits of implementing this analytical technique, one being that it is able to provide magnitude (both size and direction) of the relationship for each of the given independent variables modelled [63]. Further, logistic regression has the ability to handle both continuous (e.g. height, speed, time) and categorical (e.g. win or loss and home or away) independent variables, enabling the integration of larger, diverse datasets, which is common in elite-level sport [63]. However, like many of the other methods described in this review, it does require nuanced interpretation. Additionally, logistic regression models are preferable to use with large datasets, as this reduces the likelihood of modelling error through overfitting [63].

Demonstrating its utility in high-performance sport, Gollan et al. [64] modelled the interactions between different playing styles and match contexts (match location, opposition quality, and combined effects of both) in the English Premier League. The authors identified that irrespective of match location (home or away), teams were more likely to demonstrate an established offence and set pieces when they encountered weaker opposition [64]. Conversely, weaker opposition were less likely to play this same style when competing against their stronger counterparts—emphasising the importance of understanding the tendencies of opposing teams, such that effective game plans can be designed to counter them [64]. Similarly, Parmar et al. [8] highlighted the ability of logistic regression to model the probability of team success within Rugby League using performance indicators clustered via principal component analysis. Their results noted a 91% probability of winning if a team was able to outperform their opponent in a series of grouped performance indicators. Practically, presenting such information to coaches could support the development of match strategies that attempt to exploit the styles of play most likely leading to a win. Interestingly, logistic regression has also been used to guide training planning and periodisation by modelling the difficulty of teams’ playing schedule across the course of a competitive season in rugby union [65, 66], while Woods et al. [67] demonstrated its utility for talent identification in junior Australian football—modelling the relationship between performance in various skill tests and team association. Thus, collectively, such work demonstrates the diverse use of logistic regression in the sports performance analysis literature—ranging from modelling styles of play, supporting the planning and periodisation of practice, to assisting with talent identification, while in different sports, each of these themes are important in professional Rugby League and are topics that a developing sports performance analyst can assist with. In reference to this, the next section of this paper exemplifies each of these techniques, who have been applied to key questions in Rugby League. Thus, it is hoped that these examples can offer aspiring and developing performance analysts working in Rugby League (or other sports) guidance when seeking to resolve similar questions and analyses.

## Part 2: Case Examples

### Case Example 1: Are There Identifiable Playing Styles in the National Rugby League and are These Affected by Playing Away or at Home?

#### Introduction

The growth of systems thinking within team sport has increased levels of interest regarding the examination of collective behaviours and playing styles [68]. Broadly speaking, playing style, in team sports like Rugby League, can be defined as an identified way of playing in different phases of the game (i.e. attack, defence, or transition) [23, 24, 69]. These styles of play are considered to be deliberate tactical patterns exhibited by teams while attacking, defending or when attempting to regain ball possession [24]. Importantly, research has identified methods for resolving these playing styles using match technical performance indicators [8, 23, 69]. However, these playing styles are often governed by highly complex, nonlinear interactions between players and their environment, and thus linear approaches to analysis may not suffice. Accordingly, implementing the use of analytical techniques, like those described in the first part of this paper, could be useful in resolving game styles in team sports.

In this case study, we exemplify the utility of data reduction and decision support analysis—manifest through the use of principal component analysis, logistic regression modelling and exhaustive CHAID decision trees—for the identification of team playing styles, and their subsequent importance for explaining match success in the National Rugby League (NRL). Further, we will show the impact of factors, such as match location, on the identified playing styles.

## Methodology

Data were collected from the first 10 rounds of the 2021 NRL season. The data chosen included a selection of 25 technical performance indicators from full matches and both competing teams, in accordance with previous work [8, 35]. The data used in this example have been provided as a supplementary file for readers (Additional file 1: Appendix 1) and any additional data can be found on the following commercial website (www.nrl.com/stats/).

To identify playing styles across the sample used, principal component analysis was used to reduce the total dataset into factors. These factors have been used to identify key playing styles of teams within soccer [23, 24, 69] and the European Super League (Rugby League) [8, 31]. Thus, for the purpose of this example and like has been done elsewhere, the factors resolved here are intended to represent ‘styles of play’.

Logistic regression was then used to determine which factors were most explanatory of winning (and losing) in the NRL [8, 63]. Exhaustive CHAID was used to identify how match outcome affected team performance, using match location and the previously identified factors (playing styles) [20]. Match outcome was the dependent variable, with the first split forced for match location (home or away) to enable subsequent CHAID results to clarify how winning and losing could be explained by match location. All procedures were in accordance with ethical approval obtained from the local institutional Human Research Ethics Committee (H7968). Statistical analyses were carried out using the statistical software IBM SPSS for Windows version 25 (Armonk, NY, USA, IBM Corp.).

## Results

The results of the principal component analysis identified six factors, accounting for 73.4% of the total team performance variance across the first 10 rounds of the 2021 NRL season. In order to determine which performance indicators helped resolve which factor(s), values greater than 0.60 were extracted from the rotated component matrix (Table 1).

**Table 1:** Identified playing styles (factors) with their associated technical performance characteristics

Style	Performance indicators making up this style
‘Attacking Play’	Tries, Try Assists, Run Metres, Line breaks; Run Metres Conceded*
‘Attacking Territory’	*Runs, Tackled inside opposition *20 m*; Kick Forced Dropout; Passes*
‘Kick Returns’	*Kick Return and Kick Return Metres;*
‘Kicking’	Kicks, Kick Metres, Errors*
‘Defensive Errors’	*Line breaks Conceded, Offloads Conceded*
‘Dummy Half Runs’	*Dummy Half Runs*

*Denotes negatively correlated variable within that Factor

**Fig. 1:**
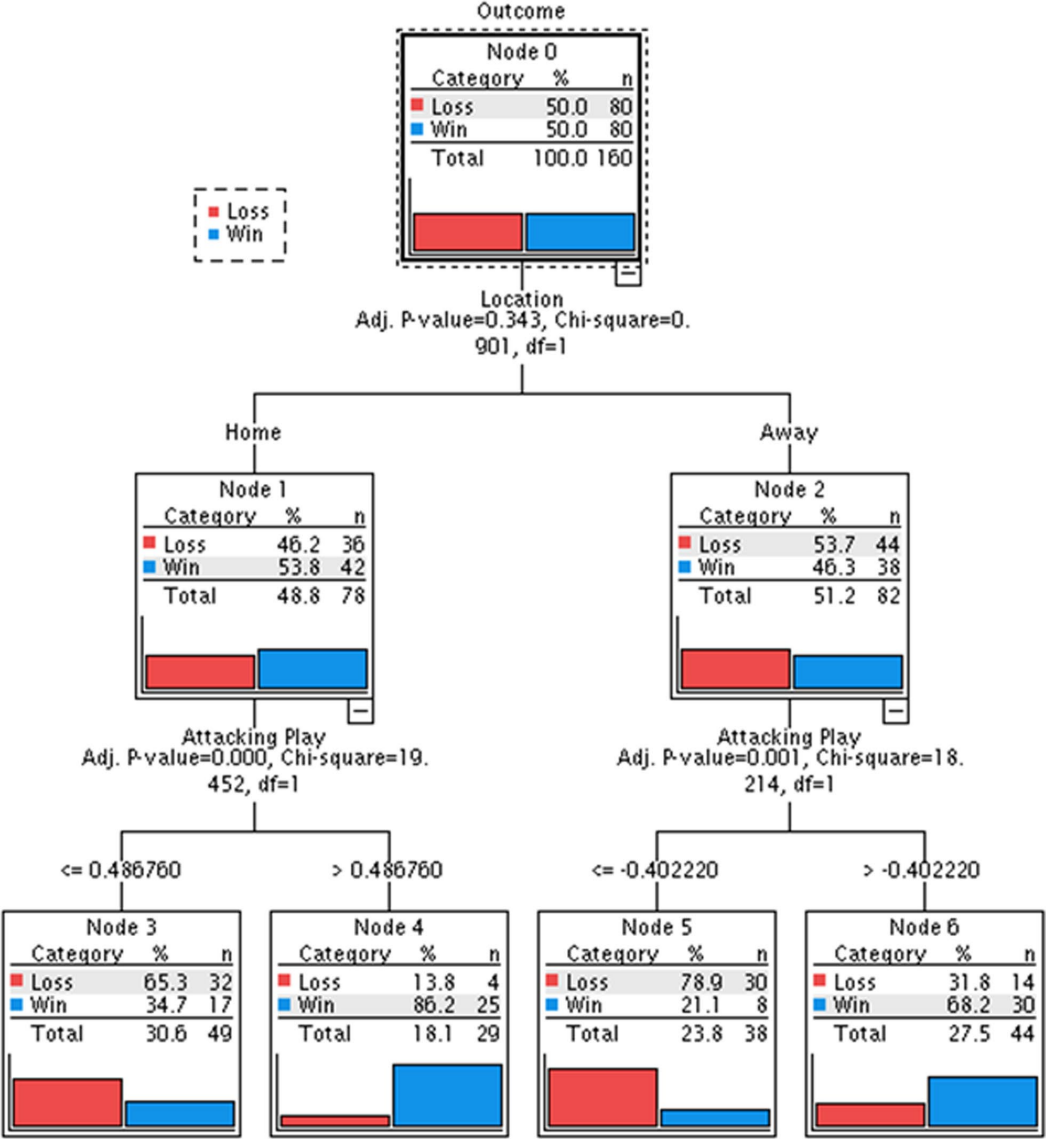
Exhaustive chi-squared automatic interaction detection (CHAID) model of match outcome as influenced by match location and team performance indicators

The logistic regression model explained 87.3% (Nagelkerke *R*^2^) of the variance of match outcome and was able to correctly classify 85.0% of all matches according to outcome. The model identified that teams were twice as likely to win when playing at home when compared to playing away (Exp(*B*) = 2.174). The exhaustive CHAID model was able to accurately classify match outcome 80% of the time using just match location and two identified styles: ‘Attacking Play’ and ‘Defensive Errors’. The visual representation of the CHAID model is presented in Fig. 1, showing that the first split of the parent Node (Node 0) was done using match location: Node 1 (Home) and Node 2 (Away). Node 1 was split by ‘Scoring’, whereby teams had a 86.2% chance of winning at home when they produced > 0.49 of the ‘Attacking Play’ component score (Node 4). Conversely teams’ likelihood of winning dropped to 34.7% (Node 3) when producing ≤ 0.49 component score for ‘Attacking Play. When playing away from home, teams that produced a component score for ‘Attacking Play’ > − 0.402 had a likelihood of winning of 68.2% (Node 6), compared with teams that had a component score ≤ − 0.402, which had a 21.1% likelihood of winning (Node 5).

## Conclusions and Practical Implications

The purpose of this case study was to exemplify for sports performance analysts a way in which they could identify playing styles in Rugby League, and how to then model these styles against outcomes like playing at or away from home. This was done using principal component analysis, logistic regression, and decision tree modelling. The principal component analysis revealed six factors, which were used as proxies of playing style, with ‘Attacking Play’, ‘Attacking Territory’, and ‘Kick Returns’ appearing most prominent over our sample period. The groups of performance indicators that made up these styles are seemingly important for distinguishing between successful and unsuccessful match performance in the NRL. Further examination of the results from the logistic regression and exhaustive CHAID models showed that both were able to correctly classify match outcome when playing at home (or away) using various playing styles > 80% of the time. These results highlight a good level of classification accuracy for both models, demonstrating the utility of either model for the identification of playing styles important for match success in the NRL. Thus, the use of analyses like those in this example can be taken by sports performance analysts to support coaches in the design of training and competition strategies that could exploit current, seemingly advantageous styles of play.

### Case Example 2: Can Individual Performance Indicators be Used to Model Playing Position Requirements in the National Rugby League?

#### Introduction

In addition to team performance, it is important to consider the varying contributions (or interactions) that may be present across the playing group (as individuals). It is these interactions, or the capabilities of playing personnel, which can be an important component of how a team performs. For example, research in Australian football [10, 61, 70], basketball [5, 39, 71], and soccer [72, 73] has identified various performance indicators that differentiate playing positions—information which can support the design and implementation of positional training and match strategies. So, how might a sports performance analyst in Rugby League identify unique playing position characteristics, used as a basis to inform operational practices like training task design or talent recruitment?

### Methods

As in the first case study, data were collected from the first 10 rounds of the 2021 NRL season. This included data for each individual for each match, which was then made relative to time played (per 80 min). All player positions were categorised a priori according to their listed playing position (player number) for that match: forward, back, spine (halves, hooker, fullback), and interchange [32]. The data used in this example have been provided as a supplementary file for readers (Additional file 2: Appendix 2), and any additional data can be found on the following commercial website (www.nrl.com/stats/).

To allow for the automatic resolution of playing positions, the dataset was first reduced into factors using principal component analysis [26, 33], with an eigenvalue of > 1 [26]. Following this, two-step cluster analysis was utilised to determine the optimal number of positional groups (clusters) through the use of the Schwartz’s Bayesian Information Criterion [74, 75]. The ‘goodness’ of the clustering was resolved by the silhouette coefficient, and additional log-likelihood distance measures were used to calculate the similarity between clusters [74, 75]. All statistical analyses were carried out using the statistical software IBM SPSS for Windows version 25 (Armonk, NY, USA, IBM Corp.).

### Results

The results of the principal component analysis identified seven factors, accounting for 70.11% of the individual performance variance across the first 10 rounds of the 2021 NRL season. In order to determine which performance indicators resolved which factor(s), values greater than 0.60 were extracted from the rotated component matrix (Table 2).

**Table 2:** Resolved factors and their associated technical performance characteristics

Factor	Performance indicators making up this factor
Factor 1 ‘Attacking’	*Runs, Run metres, Hitups, Tackled in opposition *20 m
Factor 2 ‘Kicking’	*Kicks, Kick Metres*
Factor 3 ‘Kick Returns’	*Kick Return and Kick Return Metres*
Factor 4 ‘Try Scoring’	*Tries, Line breaks*
Factor 5 ‘Errors’	Errors, Missed Tackles; Made Tackles*
Factor 6 ‘Dummy Half Passing’	*Dummy Half Runs, Passes*
Factor 7 ‘Offloads’	*Offloads Conceded, MAC Conceded*

*Denotes negatively correlated variable within that Factor

Two-step cluster analysis achieved a good silhouette measure of cohesion and separation (average silhouette = 0.7), revealing five positional classifications (clusters) in comparison to the four a priori positional groups. These positional classifications were:

- *Cluster 1 (‘Utility’):* 71% classification accuracy, 7.9% of all players, group splits as follows, 75% adjustables, 25% interchange
- *Cluster 2 (‘Interchange’):* 100% classification accuracy, 24.1% of all players
- *Cluster 3 (‘Spine’):* 100% classification accuracy, 16.2% of all players
- *Cluster 4 (‘Back’):* 99.8% classification accuracy, 19.6% of all players
- *Cluster 5 (‘Forward’):* 100% classification accuracy, 32.2% of all players

### Conclusions and Practical Implications

The purpose of this case study was to exemplify a way in which developing sports performance analysts could identify various characteristics important for different playing positions in the NRL. This was achieved using a combination of analytical methods discussed in the first section of this paper, namely principal component analysis and two-step clustering. Two-step cluster analysis revealed a fifth positional group not originally classified, identifying the positional group which could be classed as a ‘Utility’ player. This could be important information for coaches when making decisions around player recruitment and match-day interchange rotations, particularly in the event of an injury during the match to any of the spine players. Further to this point, given the recent changes to the rules in the NRL and the subsequent influence this has had on ball-in-play time and speed of the game, it is becoming more common for teams to carry a ‘utility’ type player that can cover multiple positions or be brought onto the field as an additional ball player when a team is chasing points. However, in order to determine the influence of each positional group on overall team success, further investigation would be required, possibly using some of the other approaches used in the first case study. Nevertheless, the use of analyses presented in this case study demonstrates the benefit in combining both clustering and classification approaches when seeking to understand the characteristics of different positional groups in the NRL. Further, these approaches could be used to support performance analysts with their evaluation of player performance and future positional suitability with regard to talent identification, personnel recruitment, and roster management.

## Conclusions

The growth and continued integration of sports technology can be both a blessing and a curse. For the former, it can automate the collection of data which would otherwise be laborious, yet for the latter, it can create large amounts of data that can be difficult to extract meaning from. Thus, it is important for developing performance analysts working in high-performance sport to learn *when*, *why*, and *how* to utilise various analyses to support coaches in their decision making. Thus, this paper first sought to discuss some key techniques of data reduction, clustering, decision support, and logistic regression that could be taken up by performance analysts in the field. Following this, it exemplified how such techniques could be used by sports performance analysts working in professional Rugby League.

Indeed, this paper offers a unique insight to sports performance analysis in Rugby League. It aimed, specifically, at introducing various techniques, exemplifying their use—thereby offering a basis from which developing performance analysts could begin to explore. It is envisaged that future research will follow on from the examples presented here—offering a more comprehensive insight into how techniques of data reduction, decision support analysis, and logistic regression modelling can guide various operational practices in high-performance sport.

## Supplementary Information

**Additional file 1.** Appendix 1 (file name: SMOA_CS1).

**Additional file 2.** Appendix 2 (file name: SMOA_CS2).

## Data Availability

The data used in both case examples have been provided as a supplementary file for readers (Additional file 1: Appendix 1 and Additional file 2: Appendix 2); however, all data can be found on the following commercial website (www.nrl.com/stats/). Data utilised for the case examples have been provided as a supplementary document.

## Footnotes

1 Examples of such include: University, V. Graduate Certificate in Data Analytics for Sport Performance. 2021; Available from: https://www.vu.edu.au/courses/graduate-certificate-in-data-analytics-for-sport-performance-stsp; London, M.U. Sport Performance Analysis MSc/PG Dip/PG Cert. Available from: https://www.mdx.ac.uk/courses/postgraduate/sport-performance-analysis; University, D. Graduate Certificate of Sport Performance Analysis. 2021; Available from: https://www.deakin.edu.au/course/graduate-certificate-sport-performance-analysis?_ga=2.60891180.1647635532.1626061200-1666776876.1623797527&_gac=1.21229257.1626061200.CjwKCAjwn6GGBhADEiwAruUcKoSqIcKSsOXrm08ON1SF4UTBCV57kpJeYdu34OQYdMkiJm9LFYo46xoCuq4QAvD_BwE.
